# Speech, language, functional communication, psychosocial outcomes and QOL in school-age children with congenital unilateral hearing loss

**DOI:** 10.3389/fped.2024.1282952

**Published:** 2024-03-06

**Authors:** Linda Cupples, Teresa Y. C. Ching, Sanna Hou

**Affiliations:** ^1^ Department of Linguistics, Centre for Language Sciences, Macquarie University, Sydney, NSW, Australia; ^2^ NextSense Institute, NextSense, Sydney, NSW, Australia; ^3^ Macquarie School of Education, Macquaarie University, Sydney, NSW, Australia; ^4^ School of Health and Rehabilitation Sciences, The University of Queensland, Brisbane, QLD, Australia; ^5^ National Acoustic Laboratories, Hearing Australia, Sydney, NSW, Australia

**Keywords:** unilateral hearing loss, congenital hearing loss, children, speech perception, language ability, school-age children

## Abstract

**Introduction:**

Children with early-identified unilateral hearing loss (UHL) might be at risk for delays in early speech and language, functional communication, psychosocial skills, and quality of life (QOL). However, a paucity of relevant research prohibits strong conclusions. This study aimed to provide new evidence relevant to this issue.

**Methods:**

Participants were 34 children, ages 9;0 to 12;7 (years;months), who were identified with UHL via newborn hearing screening. Nineteen children had been fitted with hearing devices, whereas 15 had not. Assessments included measures of speech perception and intelligibility; language and cognition; functional communication; psychosocial abilities; and QOL.

**Results and discussion:**

As a group, the children scored significantly below the normative mean and more than one standard deviation below the typical range on speech perception in spatially separated noise, and significantly below the normative mean on written passage comprehension. Outcomes in other aspects appear typical. There was however considerable *within participant* variation in the children's degree of hearing loss over time, raising the possibility that this pattern of results might change as children get older. The current study also revealed that participants with higher levels of nonverbal ability demonstrated better general language skills and better ability to comprehend written passages. By contrast, neither perception of speech in collocated noise nor fitting with a hearing device accounted for unique variance in outcome measures. Future research should, however, evaluate the fitting of hearing devices using random assignment of participants to groups in order to avoid any confounding influence of degree of hearing loss or children's past/current level of progress.

## Introduction

There is general agreement in the literature that the introduction of Universal Newborn Hearing Screening (UNHS) has resulted in the identification of an increased number of children with unilateral hearing loss (UHL) at an earlier age (1–3). This increase has brought with it a strengthened research focus on the impact of early identified UHL on children's language and other outcomes [e.g., (4)], and a related interest in evaluating the benefits of audiological rehabilitation with hearing aids (HAs) or cochlear implants (CIs) for this population [e.g., (5, 6)]. The aim of this research was to build on current literature; first, by examining a range of outcomes for a sample of 9-year-old children with congenital UHL; and second, by examining the association between children's outcomes and a set of predictor variables, including whether or not they had been fitted with hearing devices.

The outcome variables of direct interest in the current study were speech perception and production, language and cognition, functional auditory performance, psychosocial skills, and quality of life (QOL). Our particular focus was on children who presented with *congenital* unilateral hearing loss. A similar set of outcomes was the focus of a systematic review by Huttunen et al. (4), whose search of the literature up to February 2018 produced “no high-quality studies reporting on consequences of pre- or perilingual UHI [unilateral hearing impairment]” (p. 181). Consistent with this finding, Huttunen et al. stated that the literature they reviewed enabled them to draw “no definitive conclusions … on the impact of early-onset UHI on children's development” (p. 181). Nevertheless, individual research reports, especially those published since 2019, provide some support for the view that children with early-onset UHL achieve poorer outcomes than their age-matched peers with normal hearing (NH).

Fitzpatrick et al. (7) reported on 38 children with early-identified UHL. Thirty-five of the children presented with a congenital hearing loss, and no child was diagnosed with a severe developmental delay. The children's speech production, language, and functional auditory performance were assessed at 48 months of age, on average. When compared to a control group of age-matched children with NH, the children with UHL performed similarly on tests of receptive vocabulary and speech production, but significantly more poorly on assessments of receptive and expressive language and functional auditory performance.

Other researchers have also reported evidence of a selective impact of UHL on children's language and functional auditory outcomes. For example, Nasrallah et al. (8) reported that a group of children, ages 5–9 years, with UHL or mild bilateral HL, achieved outcomes within the average range of test normative means for receptive vocabulary, language, and speech production, but below expectations for functional auditory performance. Moreover, this pattern was true for both children with UHL and children with bilateral HL, whose scores did not differ significantly from one another. Griffin et al. (9) reported that a sample of 25 unaided children with UHL (15 congenital, ages 7;0 to 12;0 years;months) performed more poorly than a group of 14 NH children on an auditory story comprehension task when presented under challenging (noisy) conditions but not in quiet. Canẽte et al. (10) compared outcomes for a group of 12 participants, ages 7–16 years, with UHL due to congenital aural atresia, with results for 15 NH controls. Children with UHL generally performed more poorly on speech recognition in noise tasks, and especially for recognition of sentences.

Smit et al. (11) also reported on a participant sample with congenital conductive UHL due to aural atresia. Twenty-nine children and young adults, ages 6–21 years, took part in the research. Twelve of the 29 participants had an additional syndrome or medical condition, and 13 had used hearing amplification. All outcome measures were assessed using questionnaires. They included hearing QOL (in domains of spatial, speech, and quality of hearing), general QOL, language, and social-emotional-behavioural domains. The results show that study participants achieved lower scores in hearing QOL than children without hearing loss reported in the literature, and there was no effect of amplification. On the other hand, measures of general quality of life, language, and social-emotional-behavioural domains all fell within the normal range. Smit et al. (11) concluded that their study results provide evidence for a normal pattern of development in children and young adults who have conductive UHL due to aural atresia, while acknowledging that the “limited size and selection of the study population” might have contributed to their failure to detect real group differences (p. 6).

Irrespective of Smit et al.'s (11) concerns regarding possible methodological weaknesses in their study, the findings receive some support from related research. Nasrallah et al. (12) reported that a group of children, ages 5–9 years, with UHL or mild bilateral HL, achieved outcomes within the average range of test normative means for social and behavioural skills, as rated by parents and teachers. Moreover, this pattern of results was true for both children with UHL and children with bilateral HL, whose scores did not differ significantly from one another. On the other hand, findings reported by Griffin et al. (13) confirmed a significant difference in hearing-related QOL between children with UHL and those with NH.

In sum, recent studies examining the impact of UHL on children's development provide evidence of poorer outcomes relative to children with NH in functional auditory performance [e.g., (7, 8)], hearing-related quality of life [e.g., (11, 13)], and speech perception [e.g., (10, 13)]. On the other hand, non-significant differences have been observed in general QOL and psychosocial skills [e.g., (11, 12)], receptive vocabulary [e.g., (7, 8)], and speech production [e.g., (7, 8)]. With respect to language outcomes, results are inconsistent: Fitzpatrick et al. (7) found that language outcomes were worse for children with UHL compared to children with NH, whereas Nasrallah et al. (8) and Smit et al. (11) found evidence of outcomes within the typical range.

While these previously reported findings are suggestive, they do not enable strong conclusions to be drawn in regard to the impact of *congenital* UHL on children's outcomes, because most participant samples were diverse with respect to onset of hearing loss. Furthermore, children's cognitive development appears to have been overlooked in many recent published studies, despite evidence from a 2016 meta-analysis which showed that children with UHL scored significantly lower than expected on both full-scale IQ results and performance IQ (14). Hence, the first aim of the current research was to examine the impact of congenital UHL across a representative set of outcome variables including measures of speech perception and production, language and nonverbal cognition, functional auditory performance, psychosocial skills, and QOL.

The second aim of the current research was to examine the association between children's outcomes and a small set of concurrent predictor variables, which included fitting status (being fitted with a hearing device or not), nonverbal cognitive ability, and speech perception. Nonverbal ability and speech perception were included because of their demonstrated role in previous studies of speech and language outcomes achieved by children with congenital bilateral HL [e.g., (15–17)]. On the other hand, degree of hearing loss was not included as a concurrent predictor because it was not shown to play a consistent role in previous research involving children with UHL [e.g., (7, 9, 13)]. Failure to find a consistent association between degree of hearing loss and outcomes in this population might reflect, at least in part, changes in children's degree of hearing loss over time, as documented in several recent studies [e.g., (18–20)]. However, the current study was not designed to address this issue.

### The current study

The current aims were addressed in a cross-sectional study of a group of school-aged children with congenital UHL. Three primary research questions were addressed.

1. Do children with congenital UHL exhibit speech and language deficits compared to norms at school age?
2. Do children with congenital UHL exhibit functional communication deficits compared to norms at school age?
3. Do children with congenital UHL exhibit deficits in psychosocial outcomes and QOL compared to norms at school age?

In accordance with findings reported in the literature, we predicted that children with congenital UHL would achieve poorer functional auditory outcomes than expected relative to norms, but similar psychosocial outcomes and QOL. Predictions regarding speech and language outcomes were less clear, with the possibility that different outcome measures might reveal different patterns of results; for example, children with UHL might achieve similar outcomes in speech production and receptive vocabulary but poorer outcomes on speech perception and other language measures.

Two additional questions were more exploratory.

4. What might account for variation in the outcomes achieved by children with UHL (e.g., nonverbal cognitive ability, speech perception, use or not of a hearing device)?
5. Why might children who are *not fitted* with a hearing device achieve better outcomes than fitted children?

## Method

### General procedure

The protocol for this study was approved by the Australian Hearing Human Research Ethics Committee. After enrolment in the study, parents completed a questionnaire to provide demographic information, including their own level of education and any additional disabilities affecting their children. Parents also completed questionnaires soliciting information on their children's use of language and hearing in real-world environments, behavior and emotions, and QOL. Child participants completed a battery of tasks comprising audiological assessments, which were performed at the children's local hearing centres, questionnaires regarding their use of language and hearing in real-world environments, and QOL. Research speech pathologists completed direct assessments of children's spoken and written language skills and nonverbal cognitive ability. They also rated the intelligibility of children's speech. These assessments were performed at either the children's homes or hearing centres. They were conducted between age 9;0 (9 years; 0 months) and 12;7.

The definition of UHL used in this study was based on the National Workshop on Mild Bilateral and Unilateral Hearing Loss (21). In particular, UHL was defined as the average pure tone air conduction threshold at 0.5, 1, 2 kHz of any level greater than or equal to 20 dB HL or pure tone air conduction thresholds greater than 25 dB HL at two or more frequencies above 2 kHz in the affected ear with an average pure tone air conduction threshold in the good ear less than or equal to 15 dB HL.

### Participants

The current participant sample was drawn from a larger group of 153 children who were diagnosed with UHL at birth between 2002 and 2007 in New South Wales, Australia. The children were identified through Australia's nationwide newborn hearing screening program. Of the 153 children, 128 aged 9 years or older were invited to take part in the study after removing 6 children who lived remotely and a further 19 whose contact details were incomplete. Thirty-nine children and their families accepted the invitation to participate. After omitting children who subsequently withdrew from the study or did not have results available for an assessment of nonverbal cognitive ability, a final sample of 34 children remained (20F, 14M), 19 of whom were fitted with a hearing device and 15 of whom were not. Tables 1, 2 contain demographic and audiological characteristics of the final participant sample, including details of hearing devices. Just under half of the children were identified as having a disability in addition to their hearing loss (see Table 1). Disability types included: learning disability, cranio-facial abnormality, developmental delay, Golden Har syndrome, Autism Spectrum Disorder, Attention Deficit Hyperactivity Disorder, and vision problems.

**Table 1:** Demographic characteristics of participants (*N* = 34).

Characteristics	Participants (*N* = 34)	
Gender		
Male	14	41.2%
Female	20	58.8%
Age at Diagnosis in months, mean (range)	2.3	(0.4, 9.7)
Age at Assessment in years;months, mean (range)	10;4	(9;0, 12;7)
Additional Disability (excl. ANSD)		
Yes	15	44.1%
No	15	44.1%
Not specified	4	11.8%

**Table 2:** Participants’ audiological characteristics (*N* = 34).

Audiological characteristics	Participants (*N* = 34)			
Hearing Loss affected ear				
Right	17		50.0%	
Left	17		50.0%	
Type of Hearing Loss	@ diagnosis			
SNHL	19	55.9		
Conductive	9	26.5		
Mixed	2	5.9		
ANSD	2	5.9		
Not specified	2	5.9		
Degree of Hearing Loss (affected ear)^a^	@ diagnosis		@ assessment	
Typical range	0	0	5	14.7
Mild	9	26.5	6	17.6
Moderate	13	38.2	4	11.8
Severe	8	23.5	9	26.5
Profound	3	8.8	10	29.4
Not specified	1	2.9	0	0.0
Device Configuration @ assessment^b^			@ assessment	
No device			15	44.1%
15×Unilateral fitting, 4×bilateral fitting			19	55.9%

^a^ Degree of hearing loss based on a four-frequency average (0.5, 1, 2, and 4 kHz) of hearing thresholds, such that Mild <= 40 dB HL, moderate = 41–60 dB HL, severe = 61–90 dB HL, profound >= 91 dB HL.

^b^ Of 4 bilateral devices: 2 bilateral HAs + remote microphones (FMs), 1 bilateral CIs + remote microphones, 1 bilateral HAs; Of 15 unilateral devices: 5 remote microphones only, 8 unilateral HAs + remote microphones, 2 unilateral HAs only.

All children were diagnosed with hearing loss via UNHS in their first year of life [*Mean* = 2.3 months; *standard deviation (SD)* = 2.2 months]. Following diagnosis of hearing loss, all children were referred to Hearing Australia (the national government-funded hearing service provider) for audiological management, which includes ongoing hearing assessments, hearing device fitting and verification using real-ear measures according to national pediatric protocols (22).

For purposes of the current study, degree of hearing loss is expressed as mild (averaged hearing loss <=40 dB HL), moderate (41–60 dB HL), severe (61–90 dB HL) or profound (>=91 dB HL) based on a four-frequency average (0.5, 1, 2 and 4 kHz) of hearing thresholds. At diagnosis, the majority of children (61.8%) had a sensorineural or mixed hearing loss, and most (88.2%) had hearing losses in the mild to severe range. Three children (8.8%) had a profound loss at diagnosis (see Table 2). When assessments were conducted, however, the number of children with hearing losses in the profound range had increased to 10 (29.4%), and the number with mild to severe losses had dropped to 19 (55.9%). The remaining 5 children had hearing within the typical range at assessment (see Table 2). Consistent with these findings, degree of hearing loss changed from diagnosis to assessment for most individual children (*n* = 26), but most (21) of these changes involved adjacent categories (e.g., from mild to moderate or from moderate to severe). Fourteen children had a higher degree of loss at assessment than diagnosis, whereas 12 children had a higher degree of loss at diagnosis.

### Assessments

#### Audiology

Standard pure tone audiometry and tympanometry were conducted only if a child's current records were not within six months of assessment.

#### Speech and language

##### Speech perception

Speech perception was assessed using nonsense syllables (Vowel-Consonant-Vowel or VCV syllables) and sentences [Beautifully Efficient Speech Test (BEST), (23)]. Nonsense syllables were presented in collocated noise (VCV-N) at a signal-to-noise ratio (SNR) of 5 dB from a loudspeaker positioned at 0° azimuth at a distance of 0.75 metre, and performance was measured as percent correct. Sentences were presented in collocated noise at 0° azimuth (BEST-S0N0) or speech from the front at 0° and uncorrelated noise from +90° and −90° azimuth from both sides (BEST-S0N90). Performance was measured as speech reception thresholds, which were expressed in decibels (dB) SNR. Normative means and SDs for the BEST were taken from Ching et al. (24). There were no normative data available for the VCV.

##### Speech intelligibility

The Speech Intelligibility Rating scale [SIR, (25, 26)] was used to rate how easy or difficult it was to understand the children's speech. Ratings were assigned by parents and research speech pathologists (referred to as “other”) using a 6-point scale, from 1 (always understand the child with little or no effort) to 6 (almost never understand the child's speech). Normative means and SDs were obtained from a related study in our laboratory.

##### Language

The following language assessments were administered to participants by research speech pathologists.

The Peabody Picture Vocabulary Test 4th Edition [PPVT-4; (27)] is a standardized test of receptive vocabulary, using a four-alternative forced-choice, picture-pointing format in administration. It gives an overall standard score for receptive vocabulary (*Mean* = 100, *SD* = 15).

The Clinical Evaluation of Language Fundamentals—4th Edition [CELF-4; (28)] is a standardized test of spoken English (*Mean* = 100; *SD* = 15). The test includes verbal tasks which enable children to demonstrate understanding of and ability to produce English language structures. In this study an overall core language score was computed along with three subtest scores—receptive language, expressive language, and language memory.

The Woodcock Johnson III® Diagnostic Reading Battery [WJ III® DRB; (29)] comprises a set of individually administered tests, three of which were used here. Letter-word identification and word attack assessed children's ability to read aloud single words and non-words respectively; and passage comprehension assessed children's understanding of words, phrases, and/or short passages using word-picture matching and cloze procedures. The test gives an individual standard score for each test (*Mean* = 100, *SD* = 15), and a separate “Basic Reading” score, which combines results for letter-word identification and word attack.

#### Cognitive ability

Nonverbal cognitive ability was assessed using the Wechsler Nonverbal Scale of Ability [WNV; (30)], which was designed specifically for linguistically diverse populations, including people with hearing loss. This test provides a nonverbal IQ score (*Mean* = 100; *SD* = 15).

#### Functional auditory performance

The Parents' Evaluation of Aural/Oral Performance of Children [PEACH; (31)] and the Self-Evaluation of Listening Function [SELF; (32)] were used to measure children's functional auditory performance in real life. The PEACH was designed to assess children's listening and communicative behaviour in 10 real-world environments, based on observations by parents. The SELF was based on items in the PEACH with appropriate adaptations, and relied on subjective reports from children. Each item is rated on a five-point scale: never (0%), seldom (1%–25%), sometimes (26%–50%), often (51%–75%), and always (>75% of the time) by the respondent. Each assessment gives an overall score and two subscale scores, quiet and noise. Normal values for both tests were taken from a related study in our laboratory.

#### Psychosocial skills: behavior and emotions

The Strengths and Difficulties Questionnaire [SDQ; (33)] was used to assess children's behaviour and emotional difficulties. Parents completed the questionnaire, which comprises 25 items, making up five subscales: conduct problems, emotional symptoms, hyperactivity, peer relationships, and pro-social behavior. Each subscale consists of five items. The first four subscale scores (excluding prosocial behavior) were summed to make a “total difficulties score”. Australian normative data by age group (7–10 years) and gender (34) were used to calculate *z*-scores. All “difficulties” scores were reversed so that higher *z*-scores reflect less problems.

#### Quality of life (QoL)

The Pediatric Quality of Life Inventory version 4.0 Generic Core Scales (PedsQL) were used to measure children's health-related quality of life. The inventory was completed by children (PedsQL-C) and their parents (PedsQL-P). It comprises 23 items from four domains: physical functioning, emotional functioning, social functioning, and school functioning. A psychosocial health summary score was calculated as the mean score over the items answered across the emotional, social and school functioning scales. Each item is rated on a 5-point Likert scale, from 0 (never a problem) to 4 (almost always a problem). Items were reversed-scored and rescaled to a 0–100 scale, where higher scores indicate better QoL. For scale and total scores, the mean was computed as the sum across all items divided by the number of items answered. *Z*-scores were computed using normative means and SDs from Varni et al. (35).

### Data analysis

Data analysis was conducted in three stages. Stage 1 addressed the question of whether the current sample of children with congenital UHL achieved outcomes that differed from those achieved by a normative sample of children the same age. This question was addressed, first, by noting whether the mean scores achieved by the current sample were within one SD of their respective normative means; and second, using a series of 39 single-sample *t*-tests to compare the current sample's mean scores to the relevant normative means using an adjusted *α*-level of.001 (.05 ÷ 39). Stage 2 addressed the question of whether children using a hearing device would achieve different outcomes than those who did not use a hearing device. This question was addressed using a series of 41 independent samples *t*-tests with an adjusted *α*-level of .001 (.05 ÷ 41). Stage 3 addressed the question of what additional variables might account for variation in the outcomes achieved by the current sample of children. Correlational and regression techniques were used to address this question, using an *α*-level of .001 for correlations and .005 (.05 ÷ 10) for regressions.

## Results

The first three research questions asked whether school-aged children with congenital UHL would exhibit deficits compared to age-matched norms in speech and language, functional communication, psychosocial abilities, and QOL. To address these questions, mean scores were computed for all individual outcome measures across all participants. These mean scores and standard deviations are shown in Tables 3–7, along with normative values where available.

**Table 3:** Speech outcomes for the current sample: comparison with norms and fitting status.

Measure	Scale	Mean (SD)		Prob^a^	Mean (SD)		Prob^b^
		Norms	Current		Fitted	Not Fitted	
Speech perception							
VCV^c^	Quiet %	NA	96.1 (5.4)	NA	95.6 (5.9)	96.7 (5.1)	.588
	Noise %	NA	88.3 (9.9)	NA	85.9 (11.5)	91.4 (6.6)	.138
BEST^d^^,^^e^	S0N0 SNR	−1.7 (1.7)	−.85 (1.8)	.014	.02 (1.7)	−1.99 (1.1)	.001*
	S0N90 SNR	−4.5 (2.3)	**−1.11** **(4.3)**	<.001*	.78 (3.7)	−3.58 (3.9)	.004
	SRM	2.8 (1.7)	**.26** **(3.6)**	<.001*	−.76 (2.8)	1.59 (4.1)	.072
Speech production							
SIR^f^^,^^g^	Parent	1.1 (0.3)	1.3 (0.5)	.068	1.4 (0.5)	1.2 (0.4)	.199
	Other	1.2 (0.5)	1.5 (0.6)	.050	1.6 (0.7)	1.3 (0.6)	.233

NA, not available; VCV, nonsense syllables; BEST, beautifully efficient speech test; SIR, speech intelligibility rating scale. *α* = .001. Bold font is used to indicate the current group's scores that fall outside the typical range.

^a^ Probability is computed using a one-sample *t*-test comparing the current sample to norms.

^b^ Probability is computed using an independent samples *t*-test to compare children from the current sample who differ in fitting status.

^c^ *n* = 29.

^d^ *n* = 30.

^e^ BEST normative means and SDs from Ching et al. (24).

^f^ *n* = 26.

^g^ SIR norms come from a related study in our laboratory.

*p ≤ .001.

**Table 4:** Language and cognitive outcomes for the current sample: comparison with norms and fitting status.

Measure	Scale	Mean (SD)		Prob^a^	Mean (SD)		Prob^b^
		Norms	Current		Fitted	Not Fitted	
Receptive vocabulary							
PPVT SS		100 (15)	99.3 (13.5)	.772	96.6 (13.7)	102.7 (12.9)	.196
Language							
CELF4 SS	Core	100 (15)	93.9 (16.4)	.038	90.2 (16.0)	98.6 (16.2)	.141
	Rec Lang	100 (15)	91.4 (16.2)	.004	86.8 (17.0)	97.3 (13.5)	.060
	Exp Lang	100 (15)	95.9 (15.8)	.136	92.7 (15.2)	99.8 (16.2)	.200
	Lang Mem	100 (15)	94.1 (16.5)	.044	90.5 (15.7)	98.5 (17.0)	.164
Reading							
WDRB SS	Word ID	100 (15)	100.2 (14.5)	.944	97.1 (13.0)	104.1 (15.7)	.168
	Word Att	100 (15)	101.7 (11.3)	.377	98.3 (9.2)	106.1 (12.4)	.042
	Pass Comp	100 (15)	90.1 (8.1)	<.001*	88.1 (7.9)	92.6 (7.8)	.108
	Basic Read^c^	100 (15)	101.1 (13.3)	.636	97.6 (11.2)	105.5 (14.8)	.088
Nonverbal cognitive ability							
WNV SS	Full Scale	100 (15)	99.0 (13.2)	.653	93.7 (12.2)	105.7 (11.5)	.007

*N* = 34. PPVT, Peabody Picture Vocabulary Test 4th edition; CELF, Clinical Evaluation of Language Fundamentals 4th edition; WDRB, Woodcock-Johnson III Diagnostic Reading Battery; WNV, Wechsler Nonverbal Scale of ability. *α* = .001.

^a^ Probability is computed using a one-sample *t*-test comparing the current sample to norms.

^b^ Probability is computed using an independent samples *t*-test to compare children from the current sample who differ in fitting status.

^c^ WDRB Basic Reading scale combines Word ID and Word Attack.

*p ≤ .001.

**Table 5:** Functional auditory performance for the current sample: comparison to norms and fitting status.

Measure	Scale	Mean (SD)		Prob^a^	Mean (SD)		Prob^b^
		Norms	Current		Fitted	Not Fitted	
PEACH^c^	Quiet %	87.7 (12.8)	84.6 (14.6)	.292	82.7 (18.1)	86.5 (10.5)	.514
	Noise %	82.8 (15.1)	71.5 (20.0)	.008	68.2 (20.8)	74.9 (19.4)	.402
	Total %	85.5 (13.2)	78.1 (16.0)	.027	75.4 (17.8)	80.8 (14.3)	.404
SELF^d^	Quiet %	87.4 (10.4)	85.0 (15.2)	.385	83.1 (18.8)	87.7 (7.8)	.410
	Noise %	83.9 (13.2)	84.7 (17.5)	.806	81.7 (21.6)	88.9 (8.5)	.267
	Total %	85.7 (10.8)	84.8 (15.9)	.765	82.4 (20.0)	88.3 (6.5)	.315

PEACH, Parents’ Evaluation of Children's Aural/Oral Performance; SELF, Self Evaluation of Listening Function. Norms for PEACH and SELF are from a related study in our laboratory. *α* = .001.

^a^ Probability is computed using a one-sample *t*-test comparing the current sample to norms.

^b^ Probability is computed using an independent samples *t*-test to compare children from the current sample who differ in fitting status.

^c^ *n* = 26.

^d^ *n* = 31.

*p ≤ .001.

**Table 6:** Psychosocial outcomes—behavior and emotion—for the current sample: comparison to norms and fitting status.

Measure	Scale	Mean (SD)		Prob^a^	Mean (SD)		Prob^b^
		Norms	Current		Fitted	Not Fitted	
SDQP—*Z*	Emotional	0 (1.0)	−.12 (1.2)	.624	−.27 (1.38)	.02 (1.04)	.558
	Conduct	0 (1.0)	−.28 (1.1)	.215	−.20 (1.13)	−.35 (1.07)	.740
	Hyperactivity	0 (1.0)	−.15 (.88)	.401	−.12 (1.02)	−.18 (0.78)	.873
	Peer relations	0 (1.0)	−.01 (1.0)	.945	−.04 (1.21)	.01 (0.86)	.922
	Prosocial	0 (1.0)	.05 (.98)	.802	.19 (0.92)	−.08 (1.06)	.493
	Total^c^	0 (1.0)	−.18 (.95)	.360	−.20 (1.19)	−.16 (0.72)	.920

*N* = 25. SDQP—*Z*, Strengths and Difficulties Questionnaire *Z*-scores. *α* = .001.

^a^ Probability is computed using a one-sample *t*-test comparing the current sample to norms.

^b^ Probability is computed using an independent samples *t*-test to compare children from the current sample who differ in fitting status.

^c^ The first four subscales (emotional, conduct, hyperactivity, and peer relationships) were summed to make the Total Difficulties score.

*p ≤ .001.

**Table 7:** Quality of life (QOL) outcomes for the current sample: comparison to norms and fitting status.

Measure	Scale	Mean (SD)		Prob^a^	Mean (SD)		Prob^b^
		Norms	Current		Fitted	Not Fitted	
PEDSQLC—*Z*^c^	Physical	0 (1.0)	−.16 (0.93)	.328	−.38 (1.08)	.11 (0.62)	.141
	Emotional	0 (1.0)	−.51 (0.91)	.004	−.56 (1.08)	−.44 (0.68)	.720
	Social	0 (1.0)	−.11 (1.10)	.561	−.20 (1.10)	−.01 (1.12)	.628
	School	0 (1.0)	−.32 (0.88)	.049	−.47 (0.90)	−.12 (0.83)	.268
	Psychosoc	0 (1.0)	−.38 (1.01)	.039	−.50 (1.08)	−.24 (0.93)	.480
	Total	0 (1.0)	−.34 (1.03)	.071	−.50 (1.15)	−.13 (0.86)	.325
PEDSQLP—*Z*^d^	Physical	0 (1.0)	.18 (0.95)	.335	.33 (0.52)	.03 (1.25)	.434
	Emotional	0 (1.0)	−.57 (1.31)	.035	−.61 (1.58)	−.54 (1.02)	.897
	Social	0 (1.0)	−.15 (1.01)	.472	−.20 (1.15)	−.09 (0.91)	.778
	School	0 (1.0)	−.22 (0.90)	.231	−.28 (1.00)	−.15 (0.83)	.715
	Psychosoc	0 (1.0)	−.39 (1.11)	.087	−.46 (1.36)	−.32 (0.84)	.766
	Total	0 (1.0)	−.17 (0.93)	.349	−.15 (0.95)	−.20 (0.96)	.903

PEDSQLC—*Z*, Pediatric Quality of Life Inventory Child Self Report *Z*-scores; PEDSQLP—*Z*, Pediatric Quality of Life Inventory Parent Proxy Report *Z*-scores. Normative means and SDs from Varni et al. (35). *α* = .001.

^a^ Probability is computed using a one-sample *t*-test comparing the current sample to norms.

^b^ Probability is computed using an independent samples *t*-test to compare children from the current sample who differ in fitting status.

^c^ *n* = 32.

^d^ *n* = 26.

*p ≤ .001.

For the most part, these data support the view that children with UHL in the current study performed at a level similar to typically developing children of the same age. The mean scores achieved by the current sample were within one SD of their respective normative means for outcomes in language and nonverbal cognition, functional auditory performance, behavior and emotion, and QOL. On the other hand, children performed outside the typical range on speech perception in noise, when speech and noise were spatially separated. The mean SNR for the BEST S0N90 was −1.11 dB for the current group, above the expected range of −6.8 to −2.2 dB; and the observed spatial release from masking (SRM) was 0.26 dB for the current group, below the expected range of 1.1–4.5 dB (Table 3).

This pattern of results was confirmed for the most part using a series of single-sample *t*-tests to compare the current sample's mean scores with the corresponding normative means. Using a corrected *α*-level of.001 (.05 ÷ 39 individual comparisons), three differences reached statistical significance. They were the results for BEST S0N90 and BEST SRM, which confirmed our previous analysis; and the result for WDRB Passage Comprehension (*t*[33] = 7.15, *p* < .001) on which children with UHL underperformed relative to norms (see Table 4).

**Figure 1:**
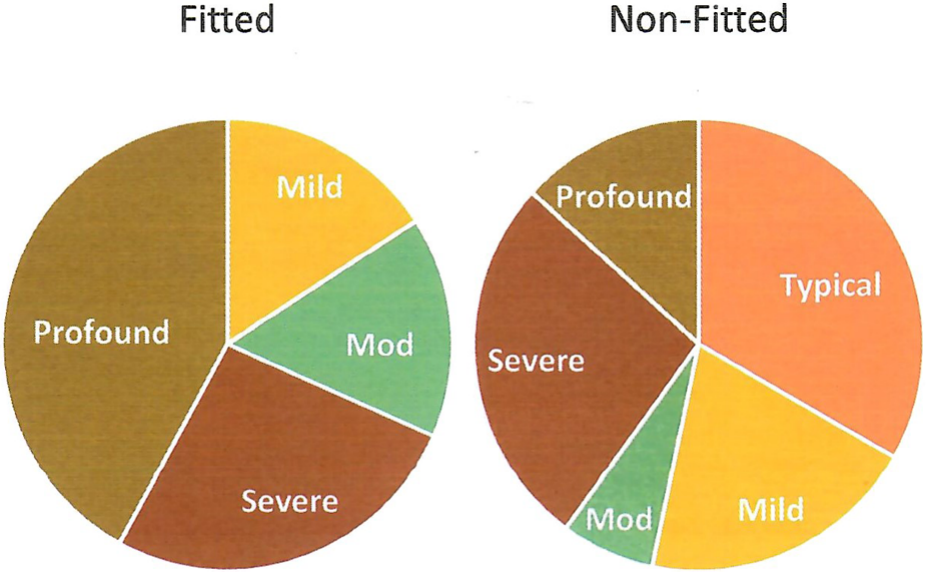
Percentage of fitted vs. non-fitted participants with different degrees of hearing loss at assessment.

Research question 4 addressed what might account for variation in the outcomes achieved by children with UHL, in particular, aspects such as cognitive ability, speech perception, and whether or not a hearing device was fitted. In a related vein, question 5 addressed why children who were *not fitted* with a hearing device might achieve better outcomes than fitted children. To shed light on these issues, children were first divided into groups according to whether they were fitted with a hearing device or not. As might be expected, these groups differed in degree of hearing loss. To illustrate, Figure 1 shows the percentage of fitted vs. non-fitted participants with different degrees of hearing loss at the time of their assessment. On the SELF questionnaire, of the 19 children with hearing devices, 2 (10.5%) had missing data, 2 (10.5%) reported using their devices 50% of the time, and 15 (78.9%) reported using their devices ≥75% of the time.

The next step was to compare the assessment outcomes achieved by fitted and non-fitted children. Mean scores were computed for all individual outcome measures for fitted vs. non-fitted participants separately. These mean scores and standard deviations are shown in Tables 3–7. Using a corrected *α*-level of.001 (.05 ÷ 41 individual comparisons), outcomes for fitted vs. non-fitted participants differ significantly on only one measure, the BEST S0N0 (*t*[28] = 3.66, *p* = .001) assessment of speech perception in collocated noise (see Table 3). This result reflected better performance (lower speech reception thresholds) in participants who had *not* been fitted with a hearing device. Although no other individual comparisons were significant, children who had *not* been fitted with a hearing device generally performed better across the range of measures than children who had been fitted.

Correlation and regression techniques provide another approach to investigate within-group variability in outcomes. For these analyses, a limited set of 10 outcome measures was used. These measures were chosen because they provided an overall reflection of performance on the various assessments. They were: SIR (other) to measure speech intelligibility; PPVT-4, CELF-4 core language, WDRB basic reading, and WDRB passage comprehension to measure language skills; PEACH total, and SELF total to measure functional auditory performance; SDQP Total to measure psychosocial skills; and PedsQL-C Total and PedsQL-P Total to measure QoL. Table 8 shows the Pearson product-moment correlations between these variables and three potential predictors: device-fitting status, WNV, and speech perception in collocated noise (BEST S0N0). Tables 9, 10 show the summary results from 10 regression analyses using these three potential predictors, one for each outcome measure.

**Table 8:** Correlations between outcome measures for the current sample.

	BEST SONO	WNV	SIR	Language				Functional auditory performance		SDQP	Quality of life	
	BEST S0N0	WNV	SIR	PPVT	CELF	WDRB Basic	WDRB Comp	PEACH	SELF	SDQP	Peds QL-C	Peds QL-P
Fitting	−.57* (30)	.46 (34)	−.24 (26)	.23 (34)	.26 (34)	.30 (34)	.28 (34)	.17 (26)	.19 (31)	.02 (25)	.18 (32)	−.03 (26)
BEST	1.00	−.24 (30)	.38 (22)	−.39 (30)	−.38 (30)	−.31 (30)	−.28 (30)	−.45 (22)	−.56 (27)	−.17 (21)	−.26 (28)	−.10 (22)
WNV		1.00	−.25 (26)	.46 (34)	.60* (34)	.54* (34)	.58* (34)	−.04 (26)	.12 (31)	.10 (25)	.51 (32)	.04 (26)
SIR			1.00	−.46 (26)	−.44 (26)	−.19 (26)	−.28 (26)	−.50 (26)	.26 (23)	−.57 (25)	−.46 (24)	−.42 (26)
PPVT				1.00	.71* (34)	.58* (34)	.78* (34)	.20 (26)	.24 (31)	.06 (25)	.31 (32)	−.05 (26)
CELF					1.00	.67* (34)	.69* (34)	.16 (26)	.41 (31)	.04 (25)	.55* (32)	.01 (26)
WDRB B						1.00	.67* (34)	.13 (26)	.37 (31)	−.02 (25)	.51 (32)	−.02 (26)
WDRB C							1.00	−.13 (26)	.23 (31)	.04 (25)	.47 (32)	−.13 (26)
PEACH								1.00	.37 (23)	.44 (25)	.17 (24)	.44 (26)
SELF									1.00	−.17 (22)	.42 (31)	−.03 (23)
SDQP										1.00	.40 (23)	.62* (25)
PedsQL-C											1.00	.44 (24)
PedsQL-P												1.00

SIR, Speech Intelligibility Ratings by research speech pathologists; CELF, CELF Core Language Score; WDRB B, WDRB Basic Reading score; WDRB C, WDRB Passage Comprehension score; PEACH, PEACH Total; SELF, SELF total; SDQP, SDQP total *Z*-score; PedsQL-C, PedsQL child report *z*-score; PedsQL-P, PedsQL parent proxy report *z*-score. *α* = .001.

*p <= .001.

**Table 9:** Regression summary table for speech and language measures (speech intelligibility, PPVT receptive vocabulary, CELF4 core language score, WDRB basic Reading, WDRB passage comprehension).

Independent variable	Outcome measure									
	SIR		Language							
	SIR		PPVT-4		CELF-4 core		WDRB basic reading		WDRB passage comprehension	
	*R* ^2^									
Fit status Y/N	.019		.057		.057		.132		.113	
BEST S0N0	.137		.097		.085		.016		.012	
WNV	.199		.180		.379 (*p* < .001)		.162		.246 (*p* = .004)	
Total *R*^2^	.355		.334		.522 (*p* < .001)		.310		.371	
*n*	22		30		30		30		30	
	Regression coefficients									
	Beta	Sig	Beta	Sig	Beta	Sig	Beta	Sig	Beta	Sig
Fit status Y/N	.526	.085	−.305	.197	−.441	.033	−.035	.882	−.123	.587
BEST S0N0	.641	.020	−.443	.033	−.449	.012	−.213	.295	−.208	.285
WNV	−.540	.030	.519	.013	.752	<.001	.492	.020	.605	.004

SIR, Speech Intelligibility ratings by research speech pathologists. Standard scores used for PPVT4, CELF-4 core language score, WDRB Basic reading, and WDRB Passage Comprehension. *α* = .005 (.05 ÷ 10).

**Table 10:** Regression summary table for functional auditory performance (PEACH, SELF), behaviour and emotion (SDQP), and quality of life (pedsQL-C, pedsQL-P).

Independent variable	Outcome measure									
	Functional auditory performance				SDQP		Quality of life			
	PEACH		SELF		SDQP		PedsQL-C		PedsQL-P	
	*R* ^2^									
Fit status Y/N	.011		.067		.002		.045		.000	
BEST S0N0	.239		.245		.031		.029		.013	
WNV	.001		.002		.010		.201		.010	
Total *R*^2^	.251		.314		.043		.275		.023	
*n*	22		27		21		28		22	
	Regression coefficients									
	Beta	Sig	Beta	Sig	Beta	Sig	Beta	Sig	Beta	Sig
Fit status Y/N	−.305	.340	−.014	.957	−.164	.649	−.229	.355	−.162	.653
BEST S0N0	−.632	.031	−.580	.010	−.255	.418	−.246	.244	−.185	.556
WNV	.043	.864	−.062	.791	.119	.684	.546	.016	.121	.673

PEACH, PEACH total; SELF, SELF total; SDQP, SDQP total *z*-score; PedsQL-C, PedsQL child report *z*-score; PedsQL-P, PedsQL parent proxy report *z*-score. *α* = .005 (.05 ÷ 10).

To summarise the correlation results: There were significant positive correlations ranging from.58 to.78 between the four language measures, indicating that children who performed well on one measure tended to score well on the other measures, as would be expected. In addition, WNV scores were positively associated with three of the four language measures (excluding PPVT), indicating that children with higher levels of nonverbal cognitive ability achieved better language outcomes. With regard to associations involving other variables, significant correlations revealed that: device fitting was associated with higher speech reception thresholds indicating poorer performance (*r* = −.57, *p* < .001); children who achieved better outcomes in CELF-4 core language also scored higher on the PedsQL-C (*r* = .55, *p* < .001); and parents' ratings of their children on the SDQP were positively associated with their ratings on the PedsQL-P (*r* = .62, *p* < .001).

Consistent with the results for the correlational analyses, multiple regressions revealed that only nonverbal cognitive ability as reflected in WNV scores accounted for significant unique variance in outcomes; in particular for CELF-4 core language and WDRB passage comprehension (see Tables 9, 10). In accordance with these results, the total variance explained in the regression analyses was generally small and non-significant. Only one regression analysis accounted for significant total variance, that of CELF-4 core language scores, with 52.2% of variance explained. The other nine analyses accounted for nonsignificant variance ranging from 2.3% to 37.1%.

## Discussion

This study was designed to investigate the outcomes achieved by a group of school-aged children with congenital UHL. Children's performance was evaluated on a comprehensive set of assessments targeting speech perception and production, language and cognition, functional auditory performance, behaviour and emotions, and QOL. The current participants achieved similar outcomes to the normative groups on all but three of the outcome measures: they required higher SNRs for speech perception in noise under conditions when speech and noise were spatially separated; they showed less spatial release from masking; and they underperformed on a test of written passage comprehension.

This pattern of results is similar in some respects to findings reported in the literature. Participants achieved typical outcomes in general QOL and psychosocial skills (behavior and emotions), consistent with previous reports by Smit et al. (11) for children and young adults ages 6–21 years, and Nasrallah et al. (12) for children ages 5–9 years. Participants also showed no marked weakness in receptive vocabulary, in accord with Fitzpatrick et al. (7) and Nasrallah et al. (8). There was limited evidence that children might exhibit a weakness in some aspects of language but not others, which might help to explain inconsistencies in findings between studies [e.g., (7, 8, 11)]. Finally, our finding that children performed below the typical range on a task assessing perception of sentences presented in noise is consistent with results described by Canẽte et al. (10), whose participant sample was similar to the current group in age (at 7–16 years of age) and congenital onset.

Setting aside these similarities, the results stand in contrast with reports in the literature that children with UHL achieve outcomes below expectations for functional auditory performance at 48 months of age (7) and 5–9 years of age (8). A possible explanation for this inconsistency across studies lies in the current study's focus on older children, of 9–12 years of age. As the data in Table 2 show, for the participants in this study, there were marked differences in degree of hearing loss across the period from diagnosis to assessment, and these changes raise the possibility that assessment results might be influenced considerably by the timepoint at which they are administered.

Another point of investigation in the current study was the identification of factors that might underlie variability in the outcomes achieved by children with UHL. As a first step, participants were allocated to groups according to whether they were fitted with a hearing device or not. Comparison of the groups' performance across the full range of outcomes revealed one significant difference: Device fitting was associated with higher speech reception thresholds indicating poorer performance. No other individual comparisons were significant, although there was a general trend in the data for fitted children to achieve worse outcomes than children who were not fitted. While this overall pattern might seem counterintuitive, it presumably reflects the fact that the decision to fit or not was influenced by children's severity of hearing loss (with aids more likely for children with more severe losses), and by how well they were progressing, that is, the decision to fit was not independent of performance, but rather, prompted partly by poor progress. The only way to ensure that results are not confounded in this way is to *randomly* assign children to participant groups according to fitting status in future studies.

Correlations and regression analyses were used to provide further evidence regarding factors that might account for variability in children's outcomes. Two factors were targeted in addition to fitting status: They were nonverbal cognitive ability and speech perception in noise. Only cognitive ability accounted for significant variance in any outcome measure, and in particular for two language measures: CELF-4 core language and WDRB passage comprehension. This finding is consistent with our previous research examining concurrent predictors of language in 5-year-old children with congenital, bilateral hearing loss (17); and more importantly, it underscores the importance of including cognitive ability in future studies of outcomes in similar participant groups.

### Strengths, limitations, and future directions

This report focuses on the outcomes achieved by a cohort of 34 children with UHL at 9 years of age. A major strength of the study lies in its inclusion of a group of children who were diagnosed with UHL at birth through Australia's universal newborn hearing screening program. By contrast, many previous studies of UHL have included more diverse groups of children with UHL and/or mild bilateral HL of varying onset. A second strength of the study lies in its use of data that were collected across a limited age range (from 9;0 to 12;7 years of age) using questionnaires and directly administered tests to assess a comprehensive set of representative outcome variables including speech production and perception, language and cognitive ability, functional auditory performance, psychosocial skills (behavior and emotions), and QOL.

Despite these strengths, the study is not without its limitations. With no longitudinal component, the results can provide only a snapshot in time with respect to children's outcomes. A longitudinal component could be particularly informative given the demonstrated variability in degree of hearing loss across time *within participants*, which is evident in the current sample and other recent investigations of children with UHL (18–20). A second limitation is the small sample size of 34, which restricted the number of independent variables that could be included in multiple regression analyses and therefore contributed to the small percentage of variance explained. Finally, as noted earlier, the effect of fitting status on outcomes was confounded in the current study because the decision to fit a hearing device was not independent of children's degree of hearing loss or their current progress.

## Conclusion

The current study investigated outcomes in 9-year speech perception and production, language and cognition, functional auditory performance, psychosocial skills (behavior and emotions), and QOL in a cohort of 34 children who were identified with UHL through Australia's universal newborn hearing screening program. As a group, the children scored significantly below the normative mean and more than one SD below the typical range on a measure of speech perception in spatially separated noise. They also scored significantly below the normative mean on written passage comprehension. Outcomes in other aspects, such as spoken language ability, psychosocial skills, QOL, and nonverbal ability appear typical. It will be important, however, to discover whether this pattern of results changes as children get older, especially in light of the within participant variation evident in the current children's degree of hearing loss over time. On a practical level, these findings enhance our understanding of the difficulties experienced by children with congenital unilateral hearing loss at school age. In particular, observed difficulties with speech perception in noise are likely to have a negative impact on children's ability to learn effectively in classrooms, which are generally noisy places. The findings in this regard underscore the importance of reducing the impact of noise in classrooms and closely monitoring children's learning on a regular basis, especially for children with unilateral hearing loss.

The current study also revealed that participants with higher levels of nonverbal cognitive ability demonstrated better general language skills and better ability to comprehend written passages. On the other hand, neither perception of speech in collocated noise nor fitting with a hearing device accounted for unique variance in outcome measures. However, further research in this area is required before strong conclusions can be drawn. For example, the effect of fitting hearing devices should include random assignment of participants to groups according to whether they are fitted or not. If random allocation is not possible, there is a strong likelihood that the decision to fit will be influenced by confounding variables, such as degree of hearing loss (children with more severe losses are more likely to be fitted with a hearing device) and past/current progress (parents of a child who is experiencing difficulties may be more likely to try something that might help than parents of a child who is doing well).

## Data Availability

The datasets presented in this article are not readily available. Individual subjects will be referred to by a subject number, which is arbitrarily selected for each subject. All data will be stored according to the National acoustic Laboratories (NAL) protocol for confidential storage. All data collected as part of the research study must be kept for at least 7 years after the publication of the findings of the study, in accordance with the requirements of the Health Privacy Principles. Paper consent forms and assessment records will be put into secure document storage at the conclusion of the study. The document storage will be managed by NAL. A scanned copy of all forms will be saved by NAL, in accordance with the requirements on data storage at NAL. Requests to access the datasets should be directed to Viji Easwar, Viji.Easwar@nal.gov.au.
